# Inhibition of the H3K4 methyltransferase SET7/9 ameliorates peritoneal fibrosis

**DOI:** 10.1371/journal.pone.0196844

**Published:** 2018-05-03

**Authors:** Ryo Tamura, Shigehiro Doi, Ayumu Nakashima, Kensuke Sasaki, Kazuya Maeda, Toshinori Ueno, Takao Masaki

**Affiliations:** Department of Nephrology, Hiroshima University Hospital, Hiroshima, Japan; Boston University Henry M Goldman School of Dental Medicine, UNITED STATES

## Abstract

Transforming growth factor-β1 (TGF-β1) is a major mediator of peritoneal fibrosis and reportedly affects expression of the H3K4 methyltransferase, SET7/9. SET7/9-induced H3K4 mono-methylation (H3K4me1) critically activates transcription of fibrosis-related genes. In this study, we examined the effect of SET7/9 inhibition on peritoneal fibrosis in mice and in human peritoneal mesothelial cells (HPMCs). We also examined SET7/9 expression in nonadherent cells isolated from the effluent of peritoneal dialysis (PD) patients. Murine peritoneal fibrosis was induced by intraperitoneal injection of methylglyoxal (MGO) into male C57/BL6 mice over 21 days. Sinefungin, a SET7/9 inhibitor, was administered subcutaneously just before MGO injection (10 mg/kg). SET7/9 expression was elevated in both MGO-injected mice and nonadherent cells isolated from the effluent of PD patients. SET7/9 expression was positively correlated with dialysate/plasma ratio of creatinine in PD patients. Sinefungin was shown immunohistochemically to suppress expression of mesenchymal cells and collagen deposition, accompanied by decreased H3K4me1 levels. Peritoneal equilibration tests showed that sinefungin attenuated the urea nitrogen transport rate from plasma and the glucose absorption rate from the dialysate. *In vitro*, sinefungin suppressed TGF-β1-induced expression of fibrotic markers and inhibited H3K4me1. These findings suggest that inhibiting the H3K4 methyltransferase SET7/9 ameliorates peritoneal fibrosis.

## Introduction

Peritoneal dialysis (PD) has been used as an effective replacement therapy for patients with end-stage kidney disease. However, long-term exposure to PD fluid leads to peritoneal fibrosis, which is clinically observed as the failure of fluid removal [1–3]. The pathogenesis of peritoneal fibrosis is characterized by loss of mesothelial cells with proliferation of α-smooth muscle actin (α-SMA)-positive myofibroblasts and deposition of extracellular matrix (ECM) proteins in submesothelial areas [4–7]. Although a number of cytokines reportedly participate in this process, transforming growth factor-β1 (TGF-β1) is considered to play a central role in the progression of peritoneal fibrosis [8–10].

Glucose is used as a hyperosmotic agent in PD fluid to enable ultrafiltration. One glucose degradation product is methylglyoxal (MGO); its level increases in response to hyperglycemia [11]. MGO is also a precursor of advanced glycation end products that damage tissues by inducing inflammation [12, 13]. Importantly, MGO levels are increased in the sera and PD fluids of PD patients [14, 15] and it reportedly plays a pivotal role in inducing peritoneal fibrosis [16–18]. In fact, peritoneal injection of MGO into rodents is a well-established method for inducing peritoneal fibrosis in animal models [19]. However, a therapeutic target in MGO-induced peritoneal fibrosis has not yet been identified.

Epigenetics is the study of gene regulatory mechanisms in which there are no alterations to DNA sequences [20, 21]. Histone post-translational modifications include acetylation, methylation, phosphorylation and ubiquitination. They regulate transcriptional activity by changing chromatin structure [22–25]. Among histone modifications, methylation of the histone tail is modulated by specific enzymes, which suggests that TGF-β1-induced histone methyltransferases are therapeutic targets for peritoneal fibrosis.

In human mesangial cells, TGF-β1 upregulates the expression of a methyl transferase, specifically, lysine 4 of histone H3 (H3K4) methyltransferase SET domain-containing lysine methyltransferase 7/9 (SET7/9). SET7/9 is responsible for the transcriptional activation of fibrotic genes [26]. We have demonstrated that inhibition of SET7/9 ameliorates renal fibrosis and decreases mono-methylation of lysine 4 in histone H3 (H3K4me1) in a mouse model of renal fibrosis [27]. These findings led us to hypothesize that sinefungin, a SET7/9 inhibitor, would suppress MGO-induced peritoneal fibrosis.

In this study, we show that SET7/9 expression in PD patients is significantly elevated compared with that in non-PD patients, and that SET7/9 expression in nonadherent cells isolated from the PD effluent is positively correlated with dialysate/plasma (D/P) ratios of creatinine (Cr) in PD patients. We also show that sinefungin alleviates both peritoneal fibrosis and peritoneal membrane dysfunction while reducing H3K4me1 expression in MGO-injected mice. Finally, we show that sinefungin suppresses both TGF-β1-induced fibrotic markers and H3K4me1 expression in primary human peritoneal mesothelial cells (HPMCs). Our resulting data suggest that sinefungin is a candidate therapeutic agent for PD patients.

## Materials and methods

### Clinical sample collection and ethics statement

To culture nonadherent cells from the PD effluent, we isolated cells from glucose-based PD fluid (1.5% Dianeal) from 12 PD patients who were treated at Hiroshima University Hospital from September 2015 to January 2017. HPMCs (described below) were used as the control. The Medical Ethics Committee of Hiroshima Graduate School of Biomedical Science approved this study (E-62), and it was performed in accordance with the Declaration of Helsinki. Written informed consent was acquired from each patient.

### Animal model

Male C57/BL6 mice (aged 10 weeks and weighing about 25 g) were obtained from Charles River Laboratories Japan (Yokohama, Japan). The mice were housed in a light- and temperature-controlled room in the Laboratory Animal Center of Hiroshima University (Hiroshima, Japan) with free access to food and water. The mice were divided into 3 groups (*n* = 5 per group): (1) the control group received intraperitoneal injections of 2.5 mL saline, (2) the MGO + saline group received intraperitoneal injections of 40 mM MGO (MP Biomedicals LLC, Illkirch, France) + subcutaneous injections of saline, (3) the MGO + sinefungin group received intraperitoneal injections of 40 mM MGO + subcutaneous injection of 10 mg/kg sinefungin (Sigma-Aldrich, St Louis, MO). Sinefungin was prepared as a suspension in saline, and administered subcutaneously (0.1 mL per mouse) just before MGO injection. We administered these solutions 5 consecutive days per week for 3 weeks. Mice were injected with 4 mL of 4.25% Dianeal solution (Baxter Health Care, Deerfield, IL, USA) to perform the PET. After 10 min, the peritoneal fluid was removed and then mice were sacrificed by cardiac puncture under deep sedation with sodium pentobarbital anesthesia. We assessed peritoneal absorption of glucose from the dialysate (D/D0) and the dialysate/plasma (D/P) ratio of urea nitrogen (UN) in the 3 groups. Parietal peritoneum samples were collected from sides contralateral to injections.

The Animal Care and Use Committee at Hiroshima University approved all of the experimental protocols (permit number: A16-61), and the experiments were performed in accordance with the National Institutes of Health Guidelines on the Use of Laboratory Animals.

### Histology and immunohistochemistry

Histologic and immunohistochemical staining of 4-μm-thick tissue sections was performed as previously described [28, 29]. The following primary antibodies were used: mouse monoclonal anti-α-SMA antibody (Sigma-Aldrich), rabbit polyclonal anti-FSP-1 antibody (Abcam, Cambridge, UK), rabbit polyclonal anti-collagen I antibody (Abcam), rabbit polyclonal anti-collagen III antibody (Abcam), rabbit polyclonal anti-TGF-β1 antibody (Santa Cruz Biotechnology, Santa Cruz, CA, USA), rabbit polyclonal anti-SET7/9 antibody (Abcam), and rabbit polyclonal anti-H3K4me1 antibody (Abcam).

Areas that contained collagens I or III were assessed in predetermined fields (×200 magnification) of the submesothelial compact zone, captured by a digital camera and analyzed using ImageJ software (version 1.48p; National Institutes of Health, Bethesda, MD, USA) in 10 fields. We counted cells expressing α-SMA, FSP-1, TGF-β1, SET7/9 or H3K4me1 in the submesothelial compact zone in 10 fields at ×200 magnification.

### Cell culture

We isolated HPMCs from human omentum as previously described [30]. The Medical Ethics Committee of Hiroshima Graduate School of Biomedical Science permitted harvesting of the omentum (E-84). Written informed consent was acquired from each patient. We maintained HPMCs in M199 medium (Life Technologies, NY, USA) including 10% fetal bovine serum (FBS) and penicillin/streptomycin. HPMCs were seeded into six-well plates and grown to subconfluence. Then, HPMCs were growth-arrested in M199 medium supplemented with 0.1% FBS for 24 h, and then treated with 5 ng/mL TGF-β1 (R &D Systems, Minneapolis, MN, USA) for 24 h. Preincubation with sinefungin (3 or 10 μg/mL) was conducted for 60 min before the 24 h of TGF-β1 stimulation. We repeated cell culture experiments five times.

### Western blotting and Enzyme-Linked Immunosorbent Assays (ELISA)

Immunoblotting and detection of secreted fibronectin were performed as previously described [31, 32]. The primary antibodies were as follows: anti-SET7/9 (Cell Signaling Technology, Danvers, MA, USA), anti-α-SMA (Sigma-Aldrich), anti-fibronectin (Sigma-Aldrich), anti-zonula occludens-1 (ZO-1; Invitrogen, Carlsbad, CA, USA), anti-α-tubulin (Sigma-Aldrich), anti-H3K4me1 (Cell Signaling Technology), and anti-H3 (Cell Signaling Technology). The intensity of each band was quantified by using ImageJ software. An ELISA kit (R&D Systems) was used to quantitate the concentrations of TGF-β1 in peritoneal fluid, following the manufacturer’s instructions.

### RNA extraction and quantitative real-time reverse transcription-PCR

RNA extraction and reverse transcription quantitative PCR were performed as previously described [33]. Specific oligonucleotide primers and probes for *ACTA2* (*α-SMA)* (assay ID: Hs00426835_g1), *Col1A2* (assay ID: Hs00164099_m1), *CTGF* (assay ID: Hs01026927_g1), *PAI-I* (assay ID: Hs01126606_m1), and *GAPDH* (assay ID: Hs02758991_g1) were obtained as TaqMan Gene Expression Assays (Applied Biosystems, Foster City, CA). *GAPDH* mRNA was used as an internal control.

### ChIP assays

Chromatin immunoprecipitation (ChIP) assays for *Col1A2* were performed using a ChIP Assay Kit (EMD Millipore, Temecula, CA, USA) as described [34]. The resulting solutions were incubated overnight at 4°C with anti-H3K4me1 antibody. DNA was purified using the QIAquick PCR Purification Kit (Qiagen, Valencia, CA, USA). Analyses of the *Col1A2* promoter region were performed by PCR reaction. The primer was designed to include the SMAD binding element (ATGCAGACA) and it was used for the amplification of the *Col1A2* promoter as follows: forward, 5′-GCGGAGGTATGCAGACAACG-3′ and reverse, 5′- GGGCTGGCTTCTTAAATTG-3′.

### Statistical analysis

Results are expressed as means ± standard deviations (S.D.). Comparisons between two groups were analyzed by Student’s *t* test. For multiple group comparisons, we used one-way ANOVA followed by *t* tests with Bonferroni corrections. Correlations were calculated by the Spearman’s rank correlation coefficient. *P* < 0.05 was considered significant.

## Results

### SET7/9 expression was elevated in mice with peritoneal fibrosis induced by MGO and was associated with functional impairment of the peritoneal membrane in PD patients

**Fig 1:**
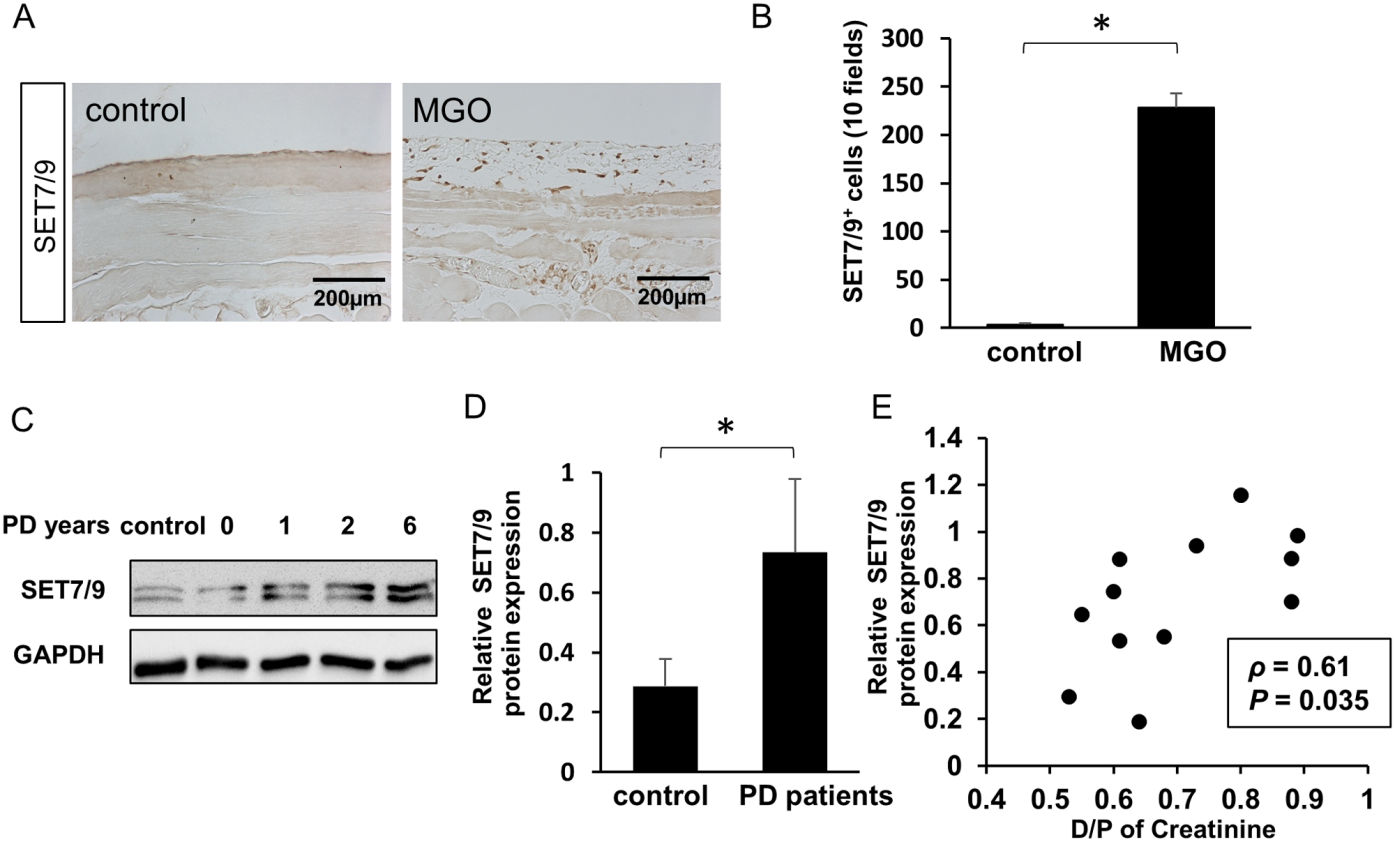
SET7/9 expression was elevated in methylglyoxal (MGO)-injected mice, and was associated with the level of functional impairment of the peritoneal membrane in PD patients’ effluents. (A) Immunohistochemical analyses of SET7/9 expression in peritoneal tissues of control- and MGO-injected mice (×200). (B) Numbers of SET7/9-positive (SET7/9^**+**^) cells in mice with or without peritoneal MGO injection (*n* = 5 for both groups). (C) SET7/9 protein expression in nonadherent cells from human PD effluents was confirmed by Western blotting. Panel: typical results. GAPDH was used as an internal control. Full-length blots are presented in S1 Fig. (D) Relative levels of SET7/9 protein expression. Controls: HPMCs from non-PD patients; PD patients: nonadherent cells isolated from PD effluent of patients who had undergone PD for ≥ 1 year. (E) Correlation between SET7/9 protein expression of nonadherent cells and the dialysate/plasma (D/P) ratio of creatinine (Cr) in PD patients (*n* = 12). Scale Bar = 200 μm. Data are means ± S.D. *, *P* < 0.05 (Student’s *t* test or Spearman’s rank correlation coefficient).

We first performed immunohistochemical staining to identify SET7/9 expression in peritoneal tissues of mice that had been injected with MGO. The number of SET7/9-positive cells in the submesothelial zone was elevated in MGO-injected mice compared with that in control mice (Fig 1A and 1B).

To evaluate the association between SET7/9 expression and peritoneal permeability, we collected nonadherent cells isolated from the PD effluent of PD patients at Hiroshima University Hospital from September 2015 to January 2017 (*n* = 12). We found that SET7/9 expression was significantly upregulated in PD patients compared with HPMCs from non-PD patients (Fig 1C and 1D). Furthermore, SET7/9 protein expression was positively correlated with D/P of Cr concentration (*ρ* = 0.61, *P* = 0.035; Fig 1E).

### Sinefungin suppressed MGO-induced peritoneal cell accumulation and thickening

**Fig 2:**
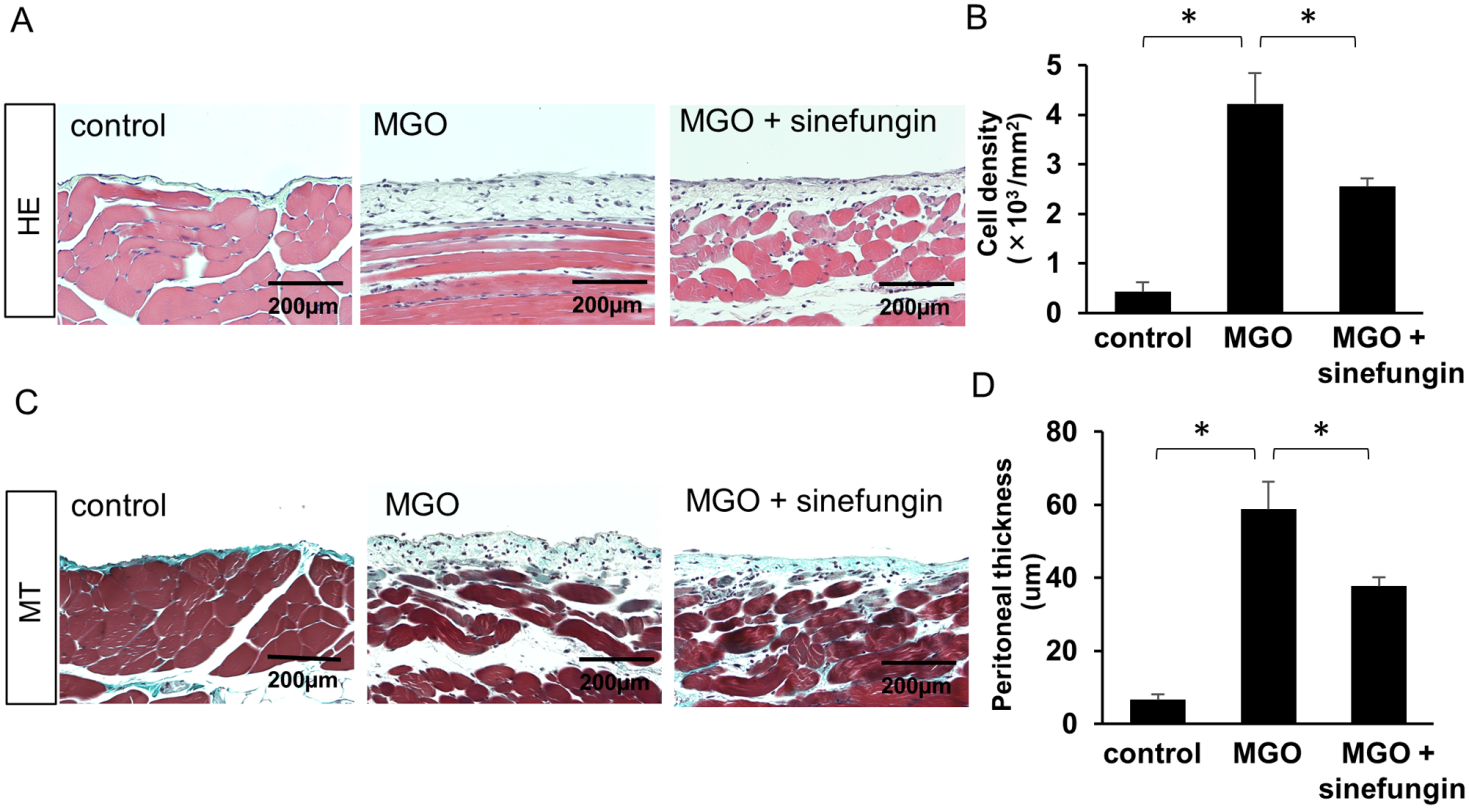
Sinefungin suppressed peritoneal cell density and thickening in MGO-injected mice. (A) Typical hematoxylin-eosin staining of peritoneal tissues of control mice, MGO-injected mice treated with vehicle only, and MGO-injected mice treated with sinefungin (×200). (B) Cell densities in the 3 groups of mice. (C) Typical Masson’s trichrome staining of peritoneal tissues of control mice, MGO-injected mice treated with vehicle only, and MGO-injected mice treated with sinefungin (×200). (D) Peritoneal thickness in the 3 groups of mice. Scale Bar = 200 μm. Data are means ± S.D. *, *P* < 0.05 (one-way ANOVA followed by *post hoc* test using *t* test with Bonferroni correction; *n* = 5 mice per group).

We conducted hematoxylin-eosin staining to evaluate changes in cell density and Masson’s trichrome staining to analyze peritoneal thickening. In mice that had been injected with MGO, peritoneal cell density increased and the submesothelial compact zone was thickened. In contrast, the subcutaneous injection of sinefungin significantly suppressed both cellularity (Fig 2A and 2B) and thickening of the submesothelial compact zone (Fig 2C and 2D) compared with vehicle-only treatment in MGO-injected mice.

### Sinefungin suppressed expression of mesenchymal markers and ECM proteins in mice with peritoneal fibrosis

**Fig 3:**
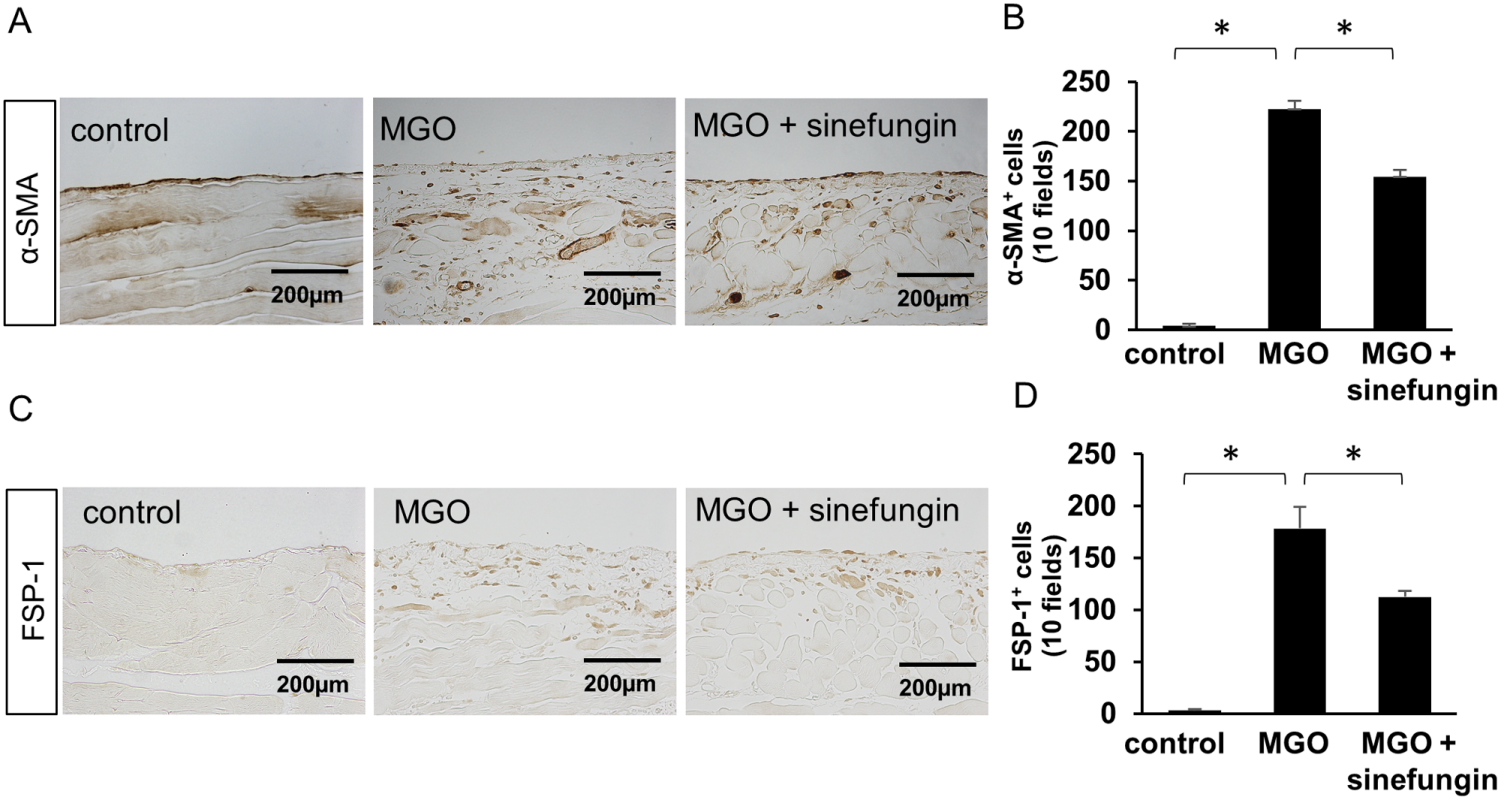
Sinefungin repressed expression of α-smooth muscle actin (α-SMA) and fibroblast-specific protein-1 (FSP-1) in mice with peritoneal fibrosis. (A) Typical α-SMA expression in peritoneal tissue of control mice, MGO-injected mice treated with vehicle only and MGO-injected mice treated with sinefungin (immunohistochemical [IHC] stain, ×200). (B) Numbers of α-SMA-positive (α-SMA^**+**^) cells in the 3 groups of mice. (C) Typical FSP-1 expression in peritoneal tissue of control mice, MGO-injected mice treated with vehicle only and MGO-injected mice treated with sinefungin (IHC stain, ×200). (D) Numbers of FSP-1-positive (FSP-1^**+**^) cells in the 3 groups of mice. The quantitative data are presented as dot plots in S2 Fig. Scale Bar = 200 μm. Data are means ± S.D. *, *P* < 0.05 (one-way ANOVA followed by *post hoc* test using *t* test with Bonferroni correction; *n* = 5 mice per group).

**Fig 4:**
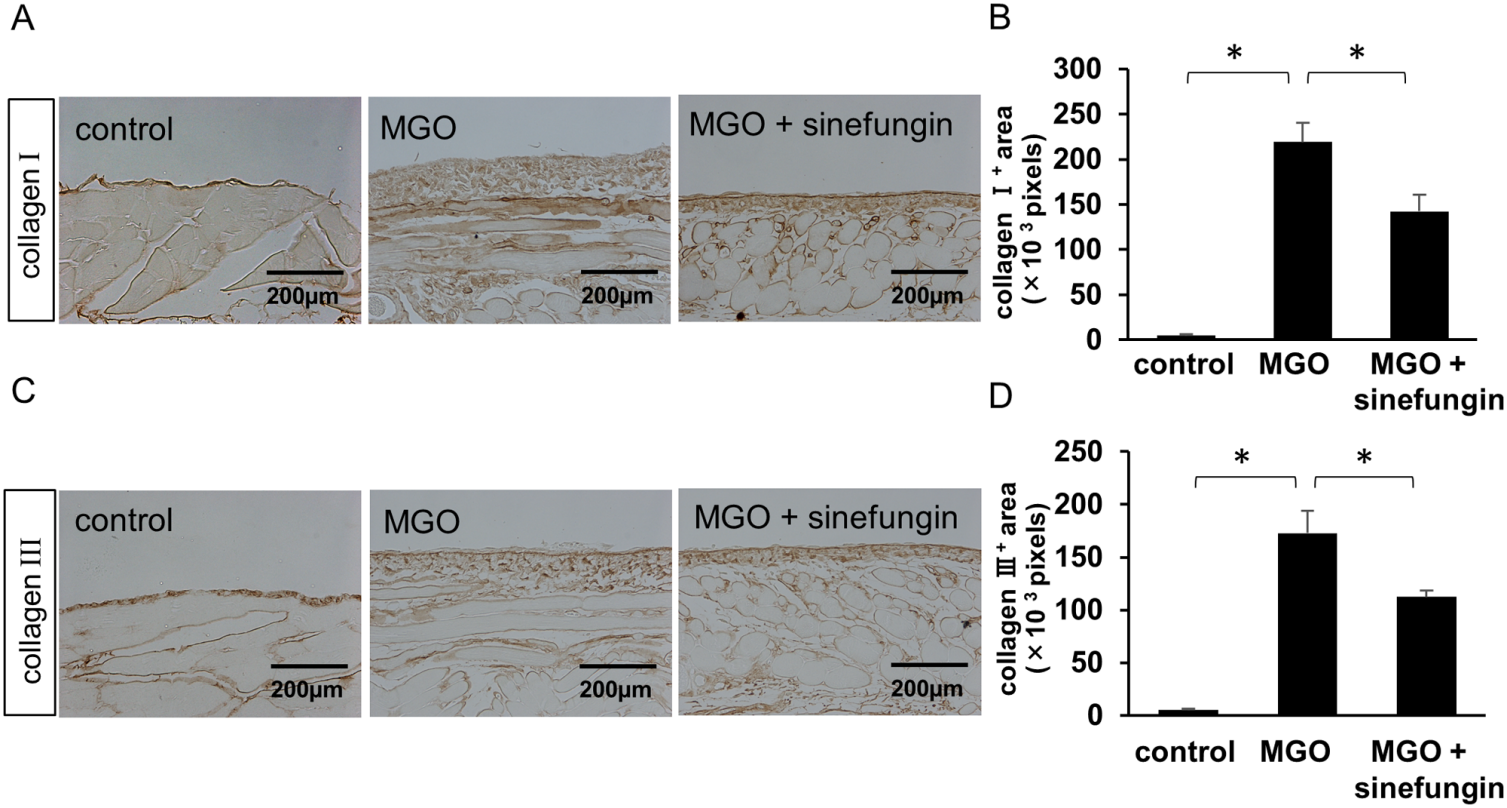
Sinefungin reduced expression of collagen types I and III in mice with peritoneal fibrosis. (A) Typical type I collagen expression in peritoneal tissue of control mice, MGO-injected mice treated with vehicle only, and MGO-injected mice treated with sinefungin (immunohistochemical [IHC] stain, ×200). (B) Numbers of type I collagen-positive (collagen I^**+**^) pixels in the 3 groups of mice. (C) Typical type III collagen expression in peritoneal tissue of control mice, MGO-injected mice treated with vehicle only, and MGO-injected mice treated with sinefungin (IHC stain, ×200). (D) Numbers of type III collagen (collagen III^+^) pixels in the 3 groups of mice. Scale Bar = 200 μm. Data are means ± S.D. *, *P* < 0.05 (one-way ANOVA followed by *post hoc* test using *t* test with Bonferroni correction; *n* = 5 mice per group).

We examined peritoneal tissues for expression of mesenchymal proteins α-SMA and FSP-1 and collagens I and III as extracellular matrix (ECM) proteins. Injections of MGO remarkably elevated α-SMA-positive myofibroblasts and FSP-1-positive cells in the submesothelial compact zone. In contrast, sinefungin significantly reduced α-SMA-positive myofibroblasts (Fig 3A and 3B) and FSP-1-positive cells (Fig 3C and 3D) compared with MGO-injected mice treated with vehicle only. The expression of collagens I and III was increased in the submesothelial compact zone of MGO-injected mice treated with vehicle only (Fig 4A and 4C). However, sinefungin significantly diminished the area in which collagens I and III accumulated in MGO-injected mice (Fig 4B and 4D).

### Sinefungin inhibited SET7/9-mediated H3K4me1 but not TGF-β1 expression

**Fig 5:**
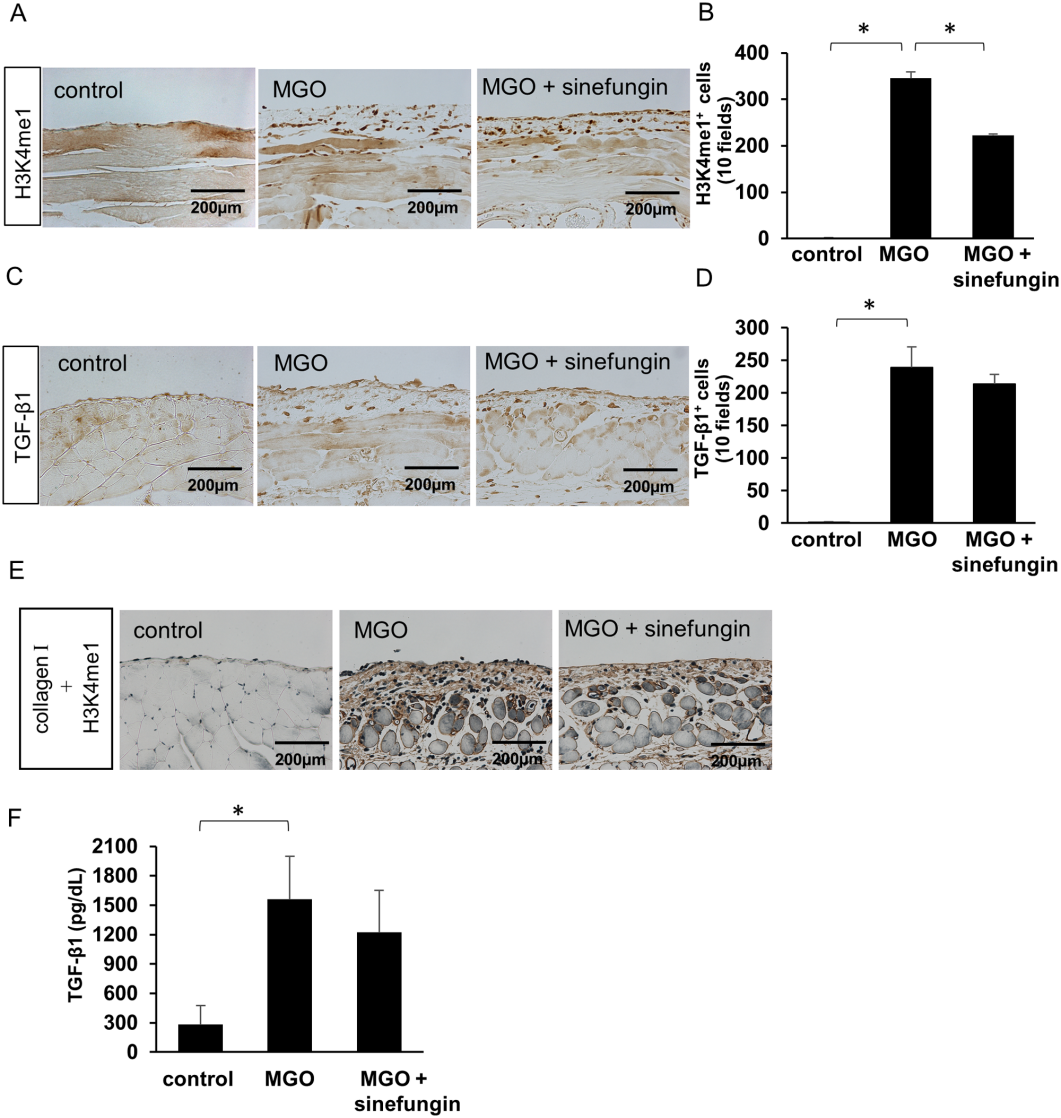
Sinefungin inhibited the expression of H3K4me1 but not that of TGF-β1 in mice with peritoneal fibrosis. (A) Typical H3K4me1 levels in peritoneal tissue of control mice, MGO-injected mice treated with vehicle only, and MGO-injected mice treated with sinefungin (immunohistochemical [IHC] stain, ×200). (B) Numbers of H3K4me1-positive (H3K4me1^**+**^) cells in the 3 groups of mice. (C) Typical TGF-β1 expression in peritoneal tissue of control mice, MGO-injected mice treated with vehicle only and MGO-injected mice treated with sinefungin (IHC stain, ×200). (D) Numbers of TGF-β1-positive (TGF-β1^**+**^) cells in the 3 groups of mice. The quantitative data are presented as dot plots in S3 Fig. (E) Two-color immunohistochemical staining showing localization of H3K4me1 (blue-gray) and collagens I (brown). (F) The concentration of TGF-β1 protein in mouse PD effluent was quantitated by ELISA. Scale Bar = 200 μm. Data are means ± S.D. *, *P* < 0.05 (one-way ANOVA followed by *post hoc* test using *t* test with Bonferroni correction; *n* = 5 mice per group).

To investigate the expression of H3K4me1 and TGF-β1 during the progression of peritoneal fibrosis, we stained tissue sections with an anti-H3K4me1 antibody and an anti-TGF-β1 antibody. In MGO-injected mice treated with vehicle only, the number of H3K4me1-positive cells in the submesothelial compact zone remarkably increased compared with control mice, whereas sinefungin significantly reduced the number of H3K4me1-positive cells (Fig 5A and 5B). The number of TGF-β1-positive cells in the submesothelial compact zone did not change in MGO-injected mice treated with sinefungin compared with those treated with vehicle only (Fig 5C and 5D). Double immunostaining simultaneously showed localization of H3K4me1 and collagen I. H3K4me1 expression increased in the areas where collagens I accumulated in MGO-injected mice that were treated with vehicle only, whereas it decreased along with collagen I expression in MGO-injected mice treated with sinefungin (Fig 5E). Similarly, sinefungin did not affect TGF-β1 protein levels in mouse peritoneal fluid (Fig 5F).

### Sinefungin reduced peritoneal membrane functional impairments in mice with peritoneal fibrosis

**Fig 6:**
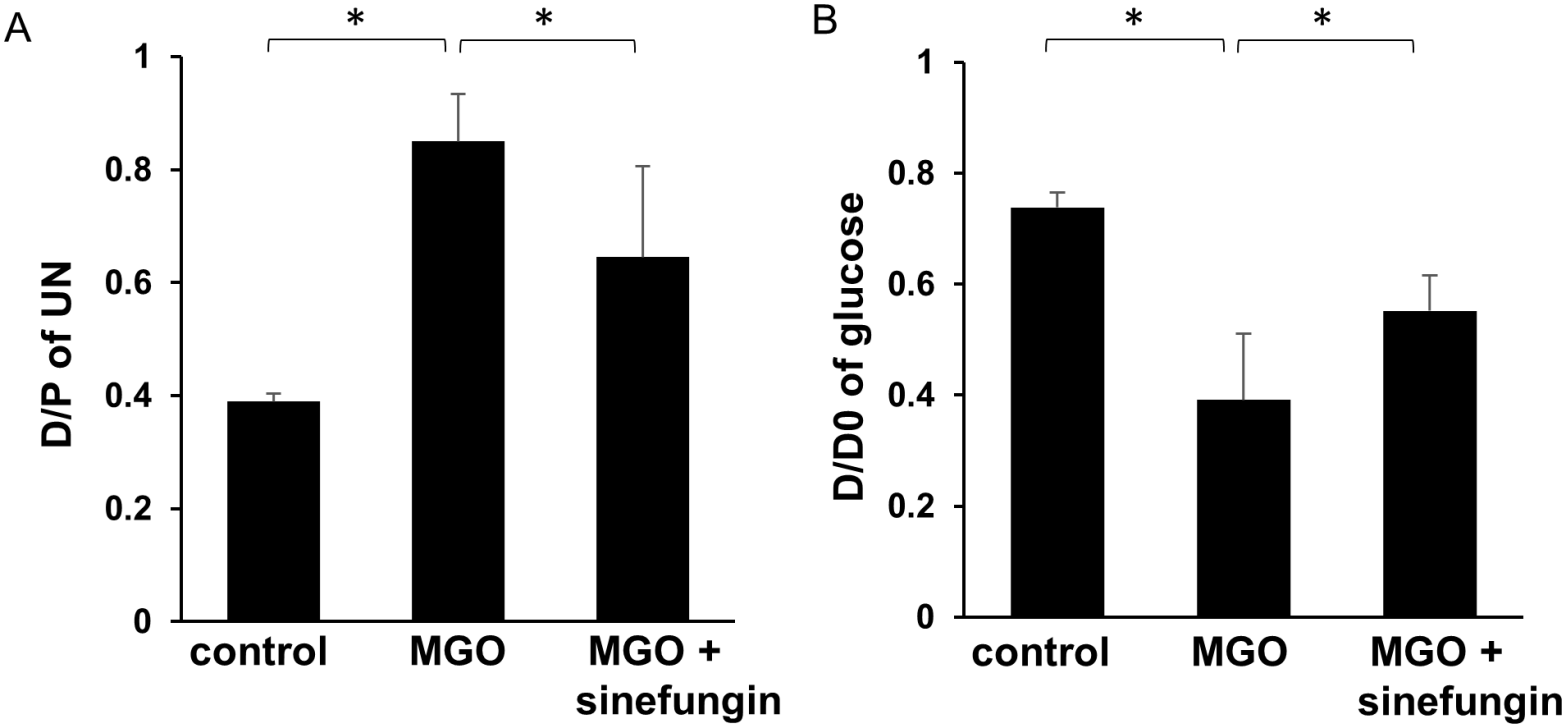
Sinefungin improved functional impairments of peritoneal membrane in mice with peritoneal fibrosis. (A) Dialysate/plasma (D/P) ratio of urea nitrogen (UN); (B) peritoneal absorption of glucose from dialysate (D/D0) in control mice, MGO-injected mice treated with vehicle only, and MGO-injected mice treated with sinefungin during 10-min dialysate dwell (4.25% dialysis solution). Data are means ± S.D. *, *P* < 0.05 (one-way ANOVA followed by *post hoc* test using *t* test with Bonferroni correction; *n* = 5 mice per group).

We performed a peritoneal equilibrium test (PET) to evaluate functional alteration of the peritoneal membrane. The urea nitrogen transport rate from plasma and the glucose absorption rate from dialysate were markedly higher in MGO-injected mice than in control mice, but these changes were significantly alleviated in MGO-injected mice treated with sinefungin (Fig 6A and 6B).

### Sinefungin inhibited TGF-β1-induced expression of fibrotic markers and H3K4me1 in HPMCs

**Fig 7:**
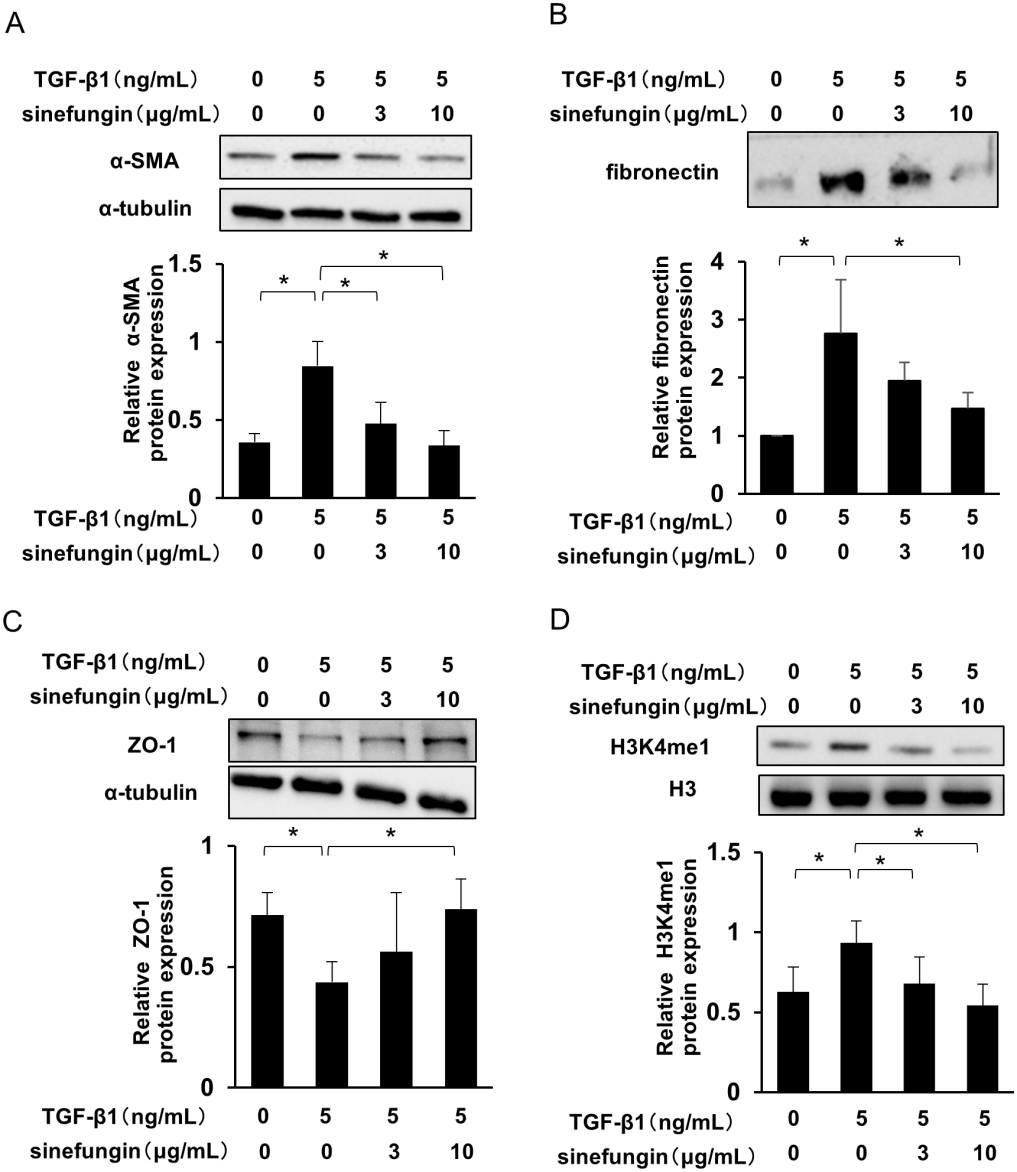
Sinefungin inhibited TGF-β1-induced fibrotic markers and H3K4me1 in HPMCs. Representative Western blotting results for the expression of (A) α-SMA (B) secreted fibronectin and (C) zonula occludens-1 (ZO-1) of HPMCs. α-tubulin was used as an internal control. Lower panel: quantification. (D) Representative Western blotting analysis showing level of H3K4me1 in HPMCs stimulated by TGF-β1. H3 was used as the internal control. Lower panel: quantification. Full-length blots are presented in S4 Fig. Data are means ± S.D. *, *P* < 0.05 (one-way ANOVA followed by the *post hoc* test using *t* test with Bonferroni correction; *n* = 5 samples per group).

**Fig 8:**
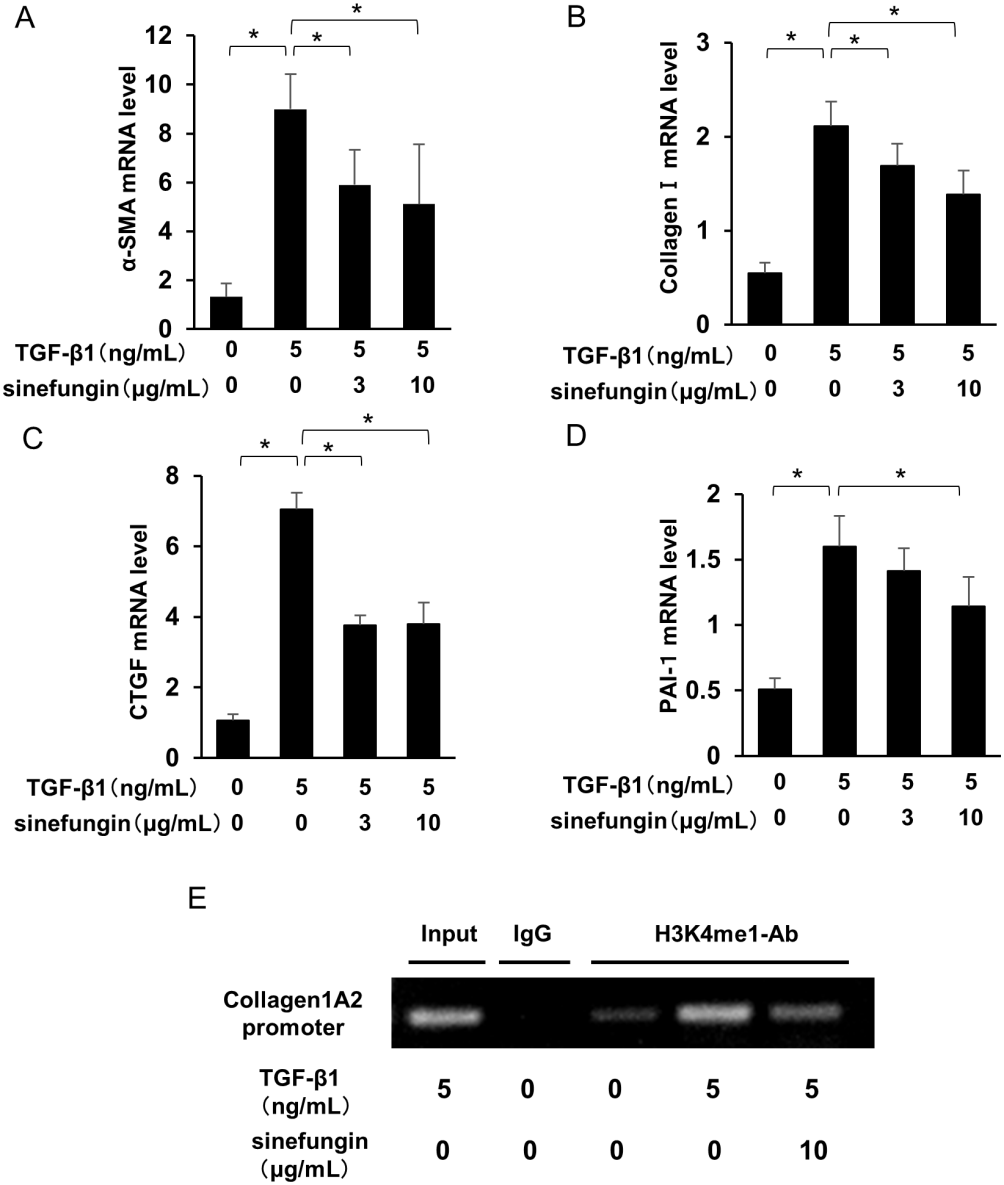
Sinefungin suppressed expression of extracellular matrix (ECM)-associated genes and H3K4me1 level at *Col1A2* promoters. Quantitative real-time polymerase chain reaction (PCR) analysis of mRNA expression of (A) *ACTA2* (*α-SMA*), (B) *Col1A2*, (C) *CTGF* and (D) *PAI-1* in HPMCs (standardized to glyceraldehyde 3-phosphate dehydrogenase [*GAPDH*]). (E) Representative chromatin immunoprecipitation (ChIP) assay of the binding of the H3K4me1 protein (H3K4me1-Ab) to *Col1A2* promoters in HPMCs. Negative control: mouse immunoglobulin G (IgG). Full-length gels are presented in S5 Fig. Data are means ± S.D. *, *P* < 0.05 (one-way ANOVA followed by the *post hoc* test using *t* test with Bonferroni correction; *n* = 5 samples per group).

TGF-β1 is an important mediator that can induce peritoneal fibrosis. To evaluate the effect of sinefungin on fibrotic changes in HPMCs, the cells were stimulated by TGF-β1, with or without sinefungin, for 24 h. TGF-β1 induced the expression of α-SMA and fibronectin, increased H3K4me1 levels and reduced ZO-1 expression. Sinefungin repressed not only these fibrotic reactions but also inhibited H3K4me1 in a dose-dependent manner (Fig 7A–7D). Likewise, preincubation of sinefungin significantly reduced mRNA expression of *ACTA2* (*α-SMA)*, *Col1*, *CTGF and PAI-1* in a dose-dependent manner (Fig 8A–8D).

We also performed chromatin immunoprecipitation (ChIP) assays of H3K4me1 in HPMCs. We found that the promoter region of the *Col1A2* was immunoprecipitated with H3K4me1 antibody. Moreover, sinefungin inhibited TGF-β1-potentiated expression of the *Col1A2* gene promoter with H3K4me1 antibody (Fig 8E).

## Discussion

In this study, we have demonstrated that sinefungin, a specific inhibitor of SET7/9, ameliorates not only peritoneal fibrosis but also peritoneal dysfunction through suppression of H3K4me1 in MGO-injected mice. Although we show that TGF-β1 induces SET7/9 expression, sinefungin does not influence TGF-β1 production in either mouse peritoneal tissue or peritoneal fluid. In *in vitro* experiments, we show that sinefungin suppresses TGF-β1-induced fibrotic markers in HPMCs and inhibits H3K4me1. Additionally, SET7/9 expression is upregulated in PD patients compared with non-PD patients, and is positively correlated with D/P of Cr concentration. These results indicate that sinefungin is a candidate therapeutic agent for PD patients to repress transcriptional activation of fibrotic genes through inhibition of H3K4me1, but not inhibition of TGF-β1 production.

Among epigenetic regulation, histone modifications, such as acetylation, methylation, phosphorylation, and ubiquitination participate in regulation of chromatin structure and transcriptional activity. Changes in histone modifications are involved in diverse diseases [35, 36]. In terms of peritoneal fibrosis, Io et al. reported that suberoylanilide hydroxamic acid, a histone deacetylase inhibitor, suppressed peritoneal fibrosis in mice through upregulation of bone morphogenetic protein (BMP)-7 [37]. Yang et al. also reported that C646, a histone acetyltransferase inhibitor attenuates peritoneal fibrosis by blocking the TGF-β1/Smad3 signaling pathway [38]. Furthermore, we previously showed that the H3K9 methyltransferase G9a is implicated in peritoneal fibrosis [39]. These findings suggest that histone modification could be a therapeutic target during peritoneal fibrosis development.

Recently, we demonstrated that inhibiting SET7/9 activity suppressed renal fibrosis and reduced the level of H3K4me1, but not that of H3K4me2 or H3K4me3 [27]. In the present study, we confirmed that sinefungin, a histone methyltransferase inhibitor, reduces TGF-β1-induced H3K4me1 levels in mice and HPMCs. Although we investigated whether MGO induced SET7/9 expression in HPMCs, MGO did not upregulate SET7/9 expression as well as subsequent H3K4 methylation (S6 Fig). Therefore, SET7/9 expression is considered to be induced through TGF-β1 expression. Furthermore, we found that the promoter region of the *Col1A2* was immunoprecipitated with H3K4me1 antibody, and that sinefungin inhibited TGF-β1-induced expression of the *Col1A2* gene promoter immunoprecipitated with H3K4me1 antibody. These findings indicate that inhibition of H3K4me1 ameliorates peritoneal fibrosis at transcriptional level of ECM proteins.

Histone lysine methyltransferases are implicated in chromatin formation, and thus in regulating gene expression [40]. Among these enzymes, SET7/9 is responsible for H3K4 methylation, which is a marker for transcriptional activation [41]. In addition to SET7/9, we also tested the possibility that TGF-β1 induces other methyltransferases, resulting in H3K4me1. As shown in S7 Fig, TGF-β1 induced the expression of SET7/9, but not SET1A, SET1B, MLL1, MLL2, or MLL4, even though all of those have been reported to induce H3K4me1 [42, 43]. Moreover, we found that SET7/9 protein expression in PD patients is significantly greater than in non-PD patients, and to be positively correlated with the D/P ratio of Cr concentration in nonadherent cells. Taken together, SET7/9 expression is a critical aspect of both peritoneal fibrosis development and peritoneal dysfunction in PD patients.

Pathologically, peritoneal fibrosis has two major features: accumulation of ECM proteins and infiltration of inflammatory cells [44]. Previous studies have demonstrated that TGF-β1 promotes fibrotic genes, and that blockading the TGF-β1 signaling pathway is the key to prevent peritoneal fibrosis [45, 46]. However, as TGF-β1 confers the ability to suppress inflammation, inhibition of TGF-β1 signaling may lead to autoimmune disease [47, 48]. In this study, inhibition of SET7/9 suppressed peritoneal fibrosis without changing TGF-β1 expression in MGO-injected mice. Since our previous studies showed that TGF-β1 is co-expressed with inflammatory cells in a mouse model of MGO-induced peritoneal fibrosis [39], the present data imply that H3K4me1 does not affect inflammation.

Regarding the mechanism by which TGF-β1 suppresses inflammation, Wakabayashi et al. found that TGF-β1 suppresses IL-2 production through H3K9me3, but not H3K4me1 [49]. Another study also showed H3K4me3 to participate in increased expression of forkhead box p3 (Foxp3), resulting in regulatory T cell (Treg) generation [50]. In fact, immunostaining for CD68-positive cells revealed that monocyte/macrophage infiltration did not differ in MGO-injected mice with or without sinfungin (S8 Fig). These results raise the possibility that sinefungin suppresses peritoneal fibrosis without disturbing inflammation.

In summary, we have demonstrated that sinefungin, an inhibitor of SET7/9, attenuates not only MGO-induced peritoneal fibrosis in mice but also TGF-β1-induced fibrotic changes in HPMCs by reducing H3K4me1 levels. We also show that SET7/9 expression in PD patients was significantly higher than in non-PD patients, and that SET7/9 expression in nonadherent cells was positively correlated with the D/P ratio of Cr concentration in PD patients. Lastly, we have clarified that the promoter of *Col1A2* is located at the H3K4me1 site. These findings indicate that SET7/9-mediated H3K4me1 could be a therapeutic target for peritoneal fibrosis.

## Supporting information

S1 Fig Uncropped image of Western blots included in Fig 1C. The red boxes indicate the cropped regions.
(TIF)

S2 Fig The quantitative data in Fig 3 are presented as dot plots. (A) Numbers of α-SMA-positive (α-SMA^**+**^) cells shown as mean ± S.D. with individual dot plots in the 3 groups of mice. (B) Number of α-SMA^+^ cells in each field of the submesothelial compact zone of all experimental mice. (C) Numbers of FSP-1-positive (FSP-1^**+**^) cells shown as mean ± S.D. with individual dot plots in the 3 groups of mice. (D) Number of FSP-1^+^ cells in each field of the submesothelial compact zone of all experimental mice. *, *P* < 0.05 (one-way ANOVA followed by *post hoc* test using *t* test with Bonferroni correction; *n* = 5 mice per group).
(TIF)

S3 Fig The quantitative data in Fig 5 are presented as dot plots. (A) Numbers of H3K4me1-positive (H3K4me1^**+**^) cells presenting mean ± S.D. with individual dot plots in the 3 groups of mice. (B) Number of H3K4me1^+^ cells in each field of submesothelial compact zone of all experimental mice. (C) Numbers of TGF-β1-positive (TGF-β1^**+**^) cells presenting mean ± S.D. with individual dot plots in the 3 groups of mice. (D) Number of TGF-β1^+^ cells in each field of the submesothelial compact zone of all experimental mice. *, *P* < 0.05 (one-way ANOVA followed by *post hoc* test using *t* test with Bonferroni correction; *n* = 5 mice per group).
(TIF)

S4 Fig Uncropped image of Western blots included in Fig 7A–7D. The red boxes indicate the cropped regions.
(TIF)

S5 Fig Uncropped image of gels included in Fig 8E. The red box indicates the cropped region.
(TIF)

S6 Fig MGO did not induce the expression of α-SMA, SET7/9, and H3K4me1 in HPMCs. Representative Western blotting results for the expression of (A) α-SMA (B) SET7/9 of HPMCs. GAPDH was used as an internal control. Lower panel: quantification. (C) Representative Western blotting analysis showing level of H3K4me1 in HPMCs. H3 was used as the internal control. Lower panel: quantification. Data are means ± S.D. *, *P* < 0.05 (Student’s *t* test; *n* = 5 samples per group).
(TIF)

S7 Fig TGF-β1 induced the expression of SET7/9, but not SET1A, SET1B, MLL1, MLL2, or MLL4 in HPMCs. Representative Western blotting results for the expression of (A) SET7/9 (B) SET1A (C) SET1B (D) MLL1 (E) MLL2 and (F) MLL4 of HPMCs. GAPDH was used as an internal control. Lower panel: quantification. Data are means ± S.D. *, *P* < 0.05 (Student’s *t* test; *n* = 5 samples per group).
(TIF)

S8 Fig Sinefungin did not affect monocyte/macrophage infiltration in mice with peritoneal fibrosis. (A) Typical CD68 expression in peritoneal tissues of control mice, MGO-injected mice treated with vehicle only and MGO-injected mice treated with sinefungin (immunohistochemical [IHC] stain, ×200). (B) Numbers of CD68-positive (CD68^+^) cells in the 3 groups of mice. Scale Bar = 200 μm. Data are means ± S.D. *, *P* < 0.05 (one-way ANOVA followed by *post hoc* test using *t* test with Bonferroni correction; *n* = 5 mice per group).
(TIF)
