# Supplementary figures and images for: Inhibition of the H3K4 methyltransferase SET7/9 ameliorates peritoneal fibrosis
Source: PLoS One. 2018 May 3;13(5):e0196844. doi: 10.1371/journal.pone.0196844 (PMC5933785; doi:10.1371/journal.pone.0196844)

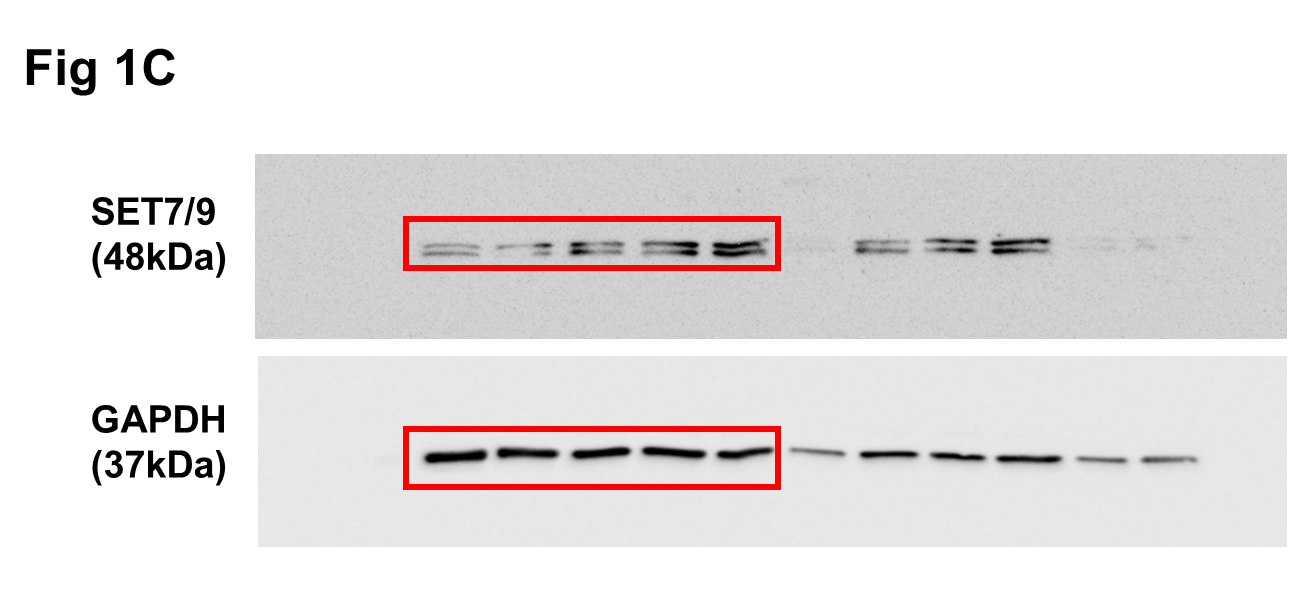

Supplement: S1 Fig — The red boxes indicate the cropped regions. (TIF) [file pone.0196844.s001.tif]

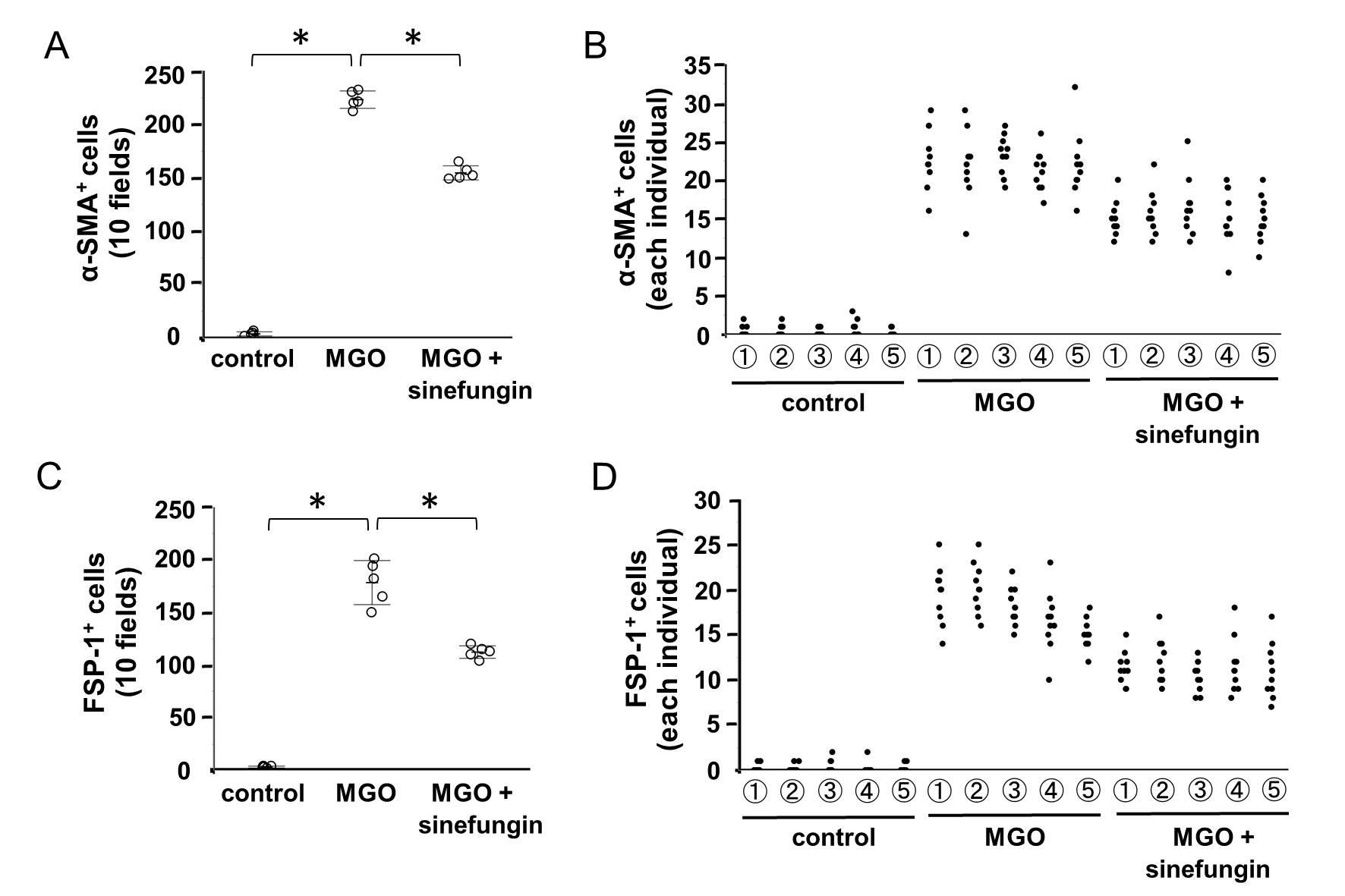

Supplement: S2 Fig — (A) Numbers of α-SMA-positive (α-SMA+) cells shown as mean ± S.D. with individual dot plots in the 3 groups of mice. (B) Number of α-SMA+ cells in each field of the submesothelial compact zone of all experimental mice. (C) Numbers of FSP-1-positive (FSP-1+) cells shown as mean ± S.D. with individual dot plots in the 3 groups of mice. (D) Number of FSP-1+ cells in each field of the submesothelial compact zone of all experimental mice. *, P < 0.05 (one-way ANOVA followed by post hoc test using t test with Bonferroni correction; n = 5 mice per group). (TIF) [file pone.0196844.s002.tif]

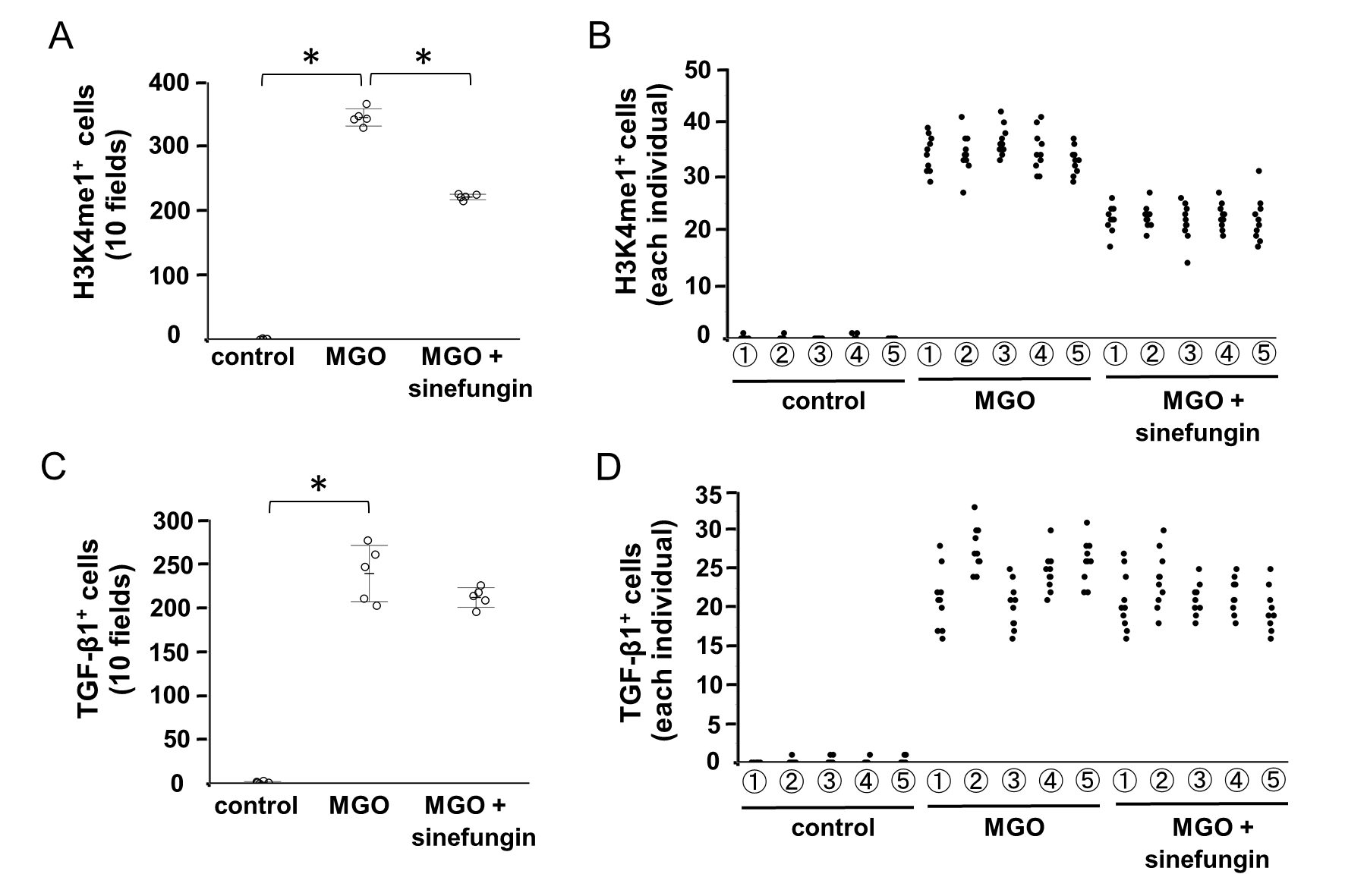

Supplement: S3 Fig — (A) Numbers of H3K4me1-positive (H3K4me1+) cells presenting mean ± S.D. with individual dot plots in the 3 groups of mice. (B) Number of H3K4me1+ cells in each field of submesothelial compact zone of all experimental mice. (C) Numbers of TGF-β1-positive (TGF-β1+) cells presenting mean ± S.D. with individual dot plots in the 3 groups of mice. (D) Number of TGF-β1+ cells in each field of the submesothelial compact zone of all experimental mice. *, P < 0.05 (one-way ANOVA followed by post hoc test using t test with Bonferroni correction; n = 5 mice per group). (TIF) [file pone.0196844.s003.tif]

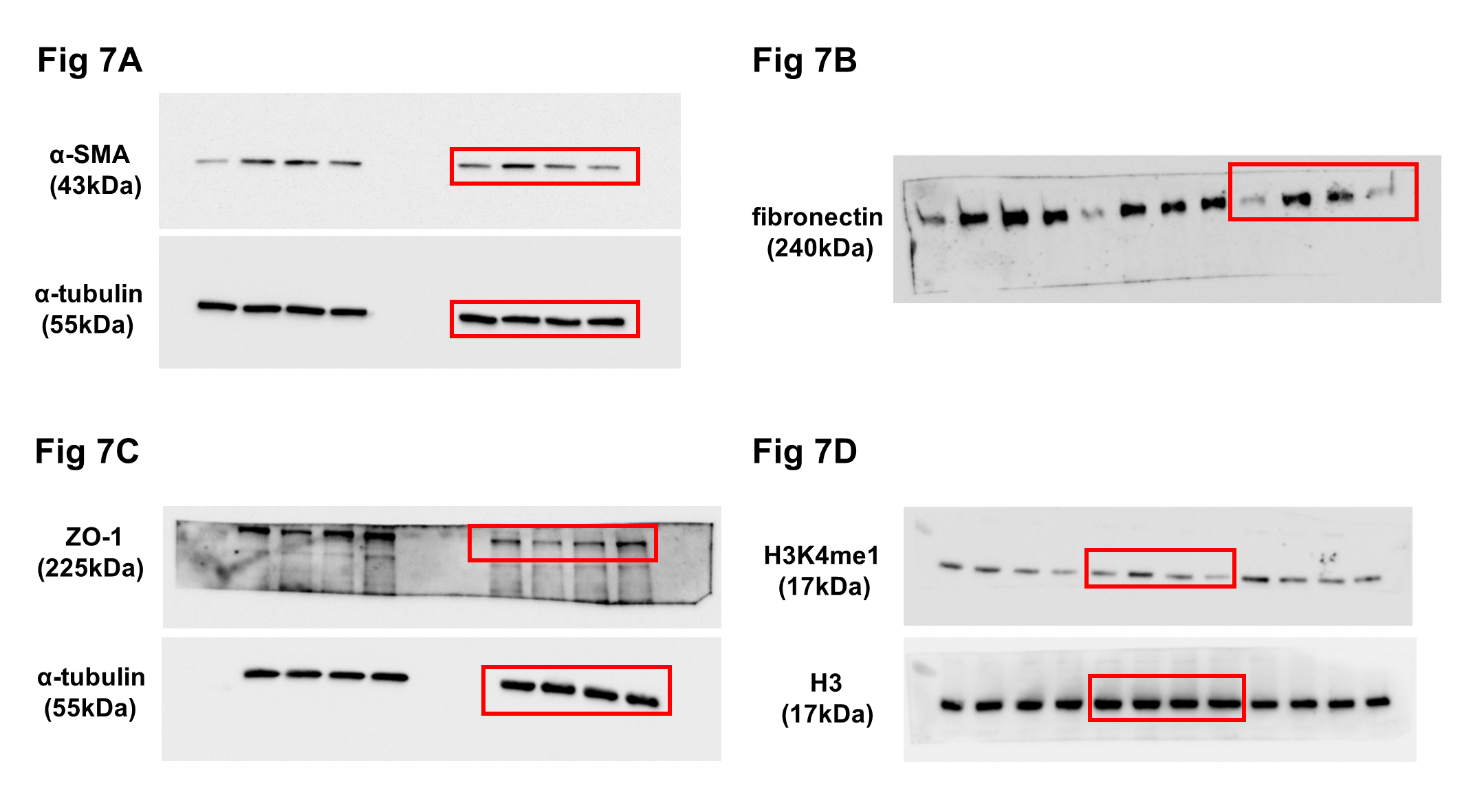

Supplement: S4 Fig — The red boxes indicate the cropped regions. (TIF) [file pone.0196844.s004.tif]

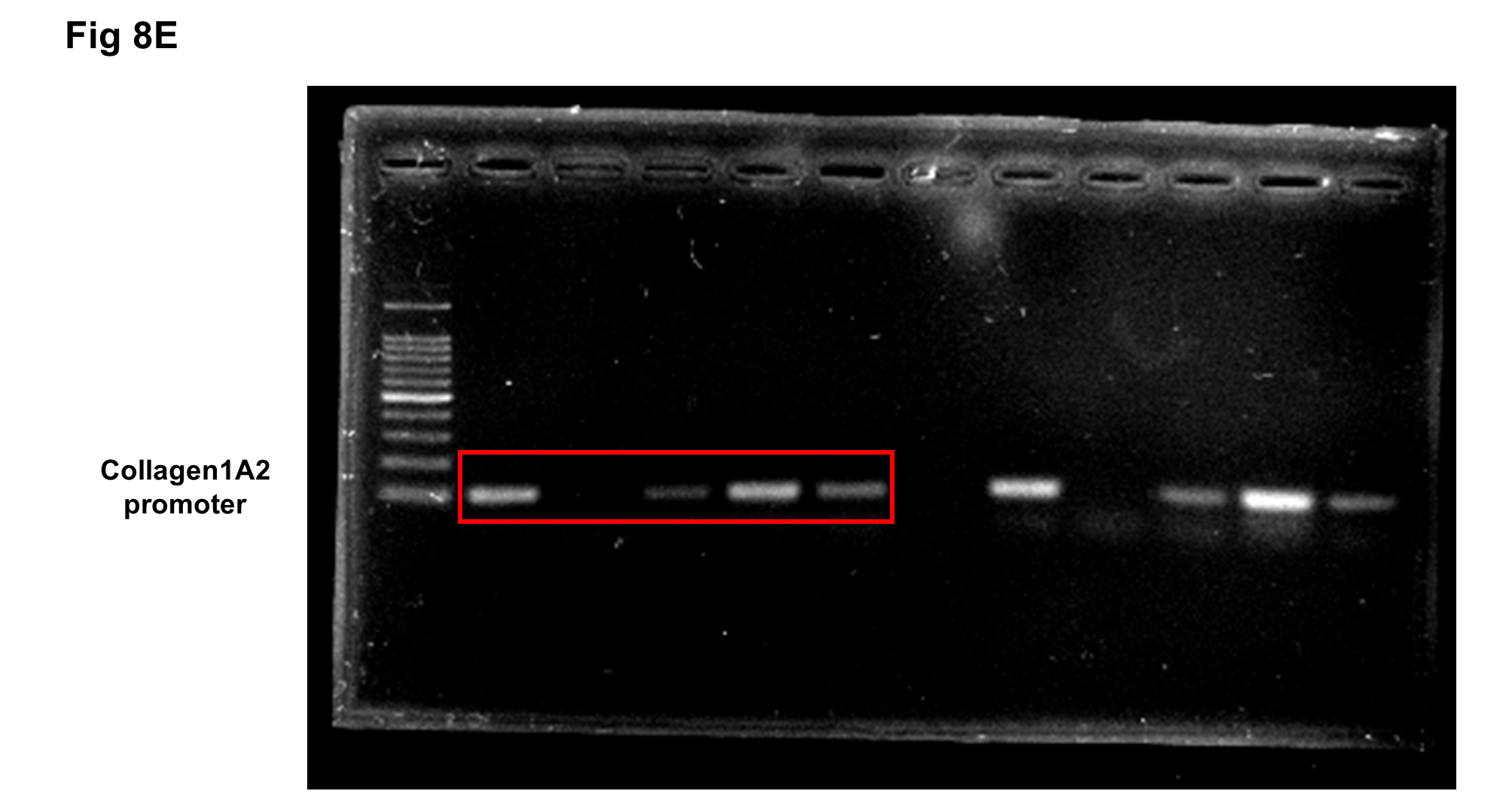

Supplement: S5 Fig — The red box indicates the cropped region. (TIF) [file pone.0196844.s005.tif]

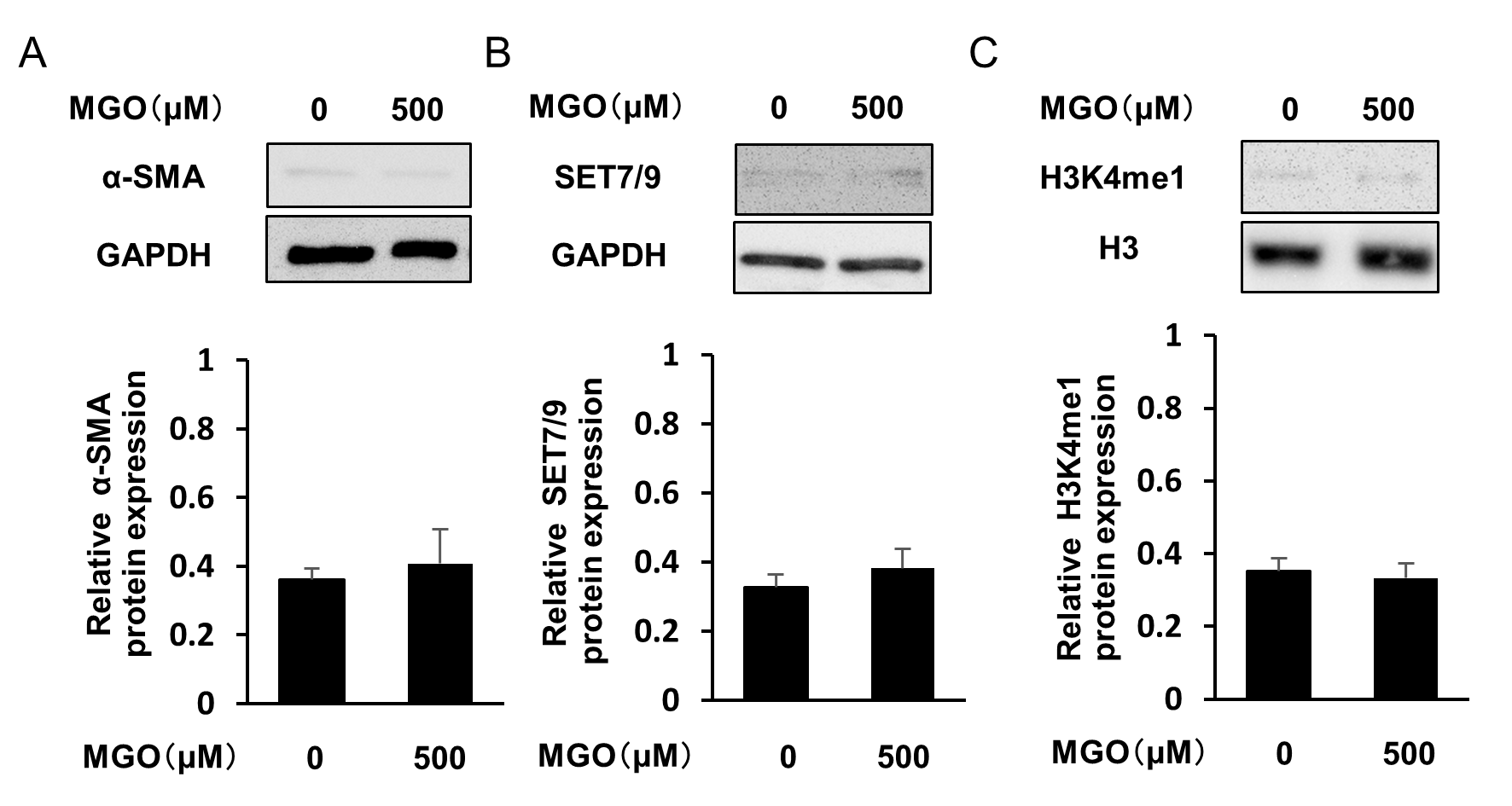

Supplement: S6 Fig — Representative Western blotting results for the expression of (A) α-SMA (B) SET7/9 of HPMCs. GAPDH was used as an internal control. Lower panel: quantification. (C) Representative Western blotting analysis showing level of H3K4me1 in HPMCs. H3 was used as the internal control. Lower panel: quantification. Data are means ± S.D. *, P < 0.05 (Student’s t test; n = 5 samples per group). (TIF) [file pone.0196844.s006.tif]

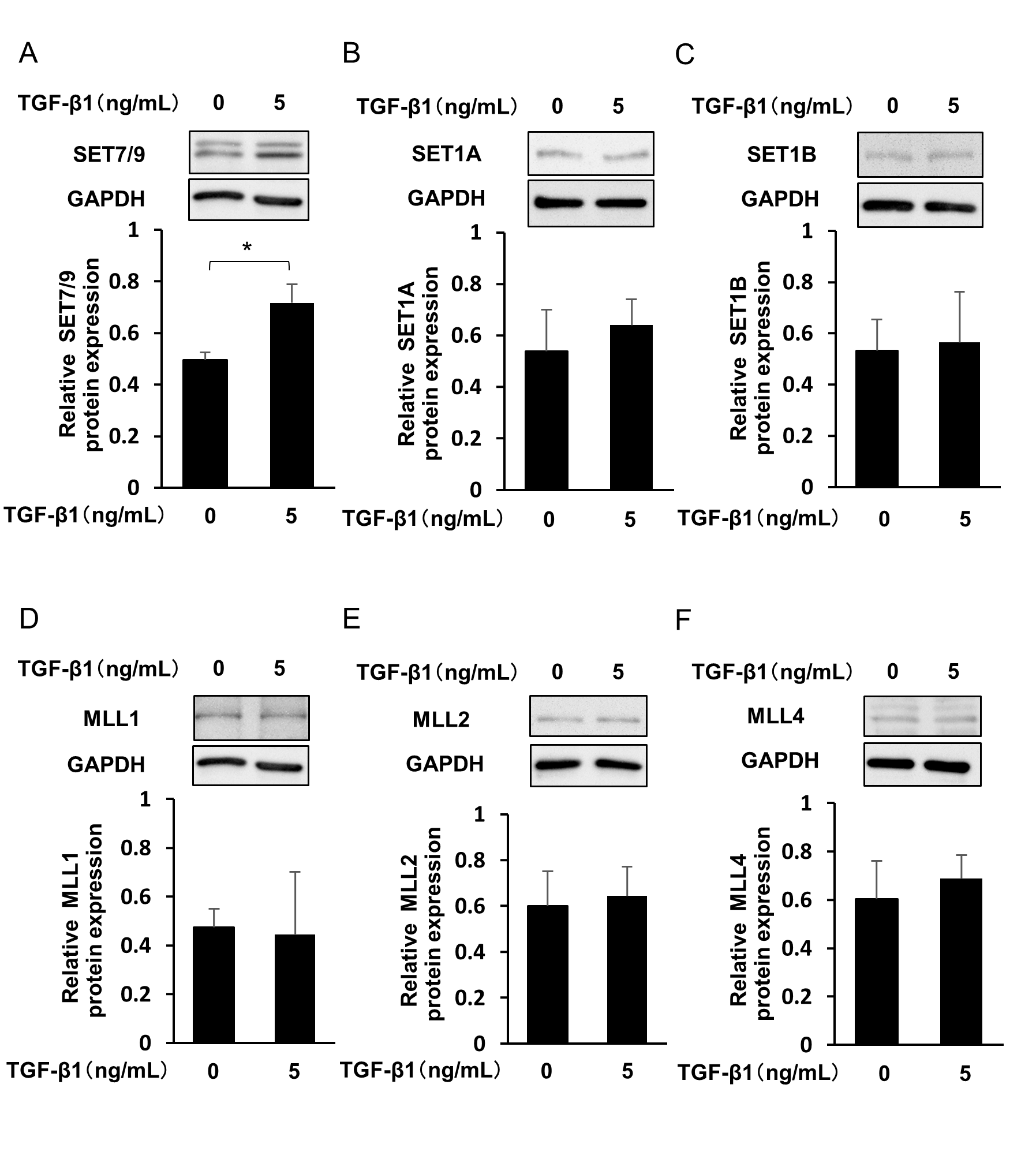

Supplement: S7 Fig — Representative Western blotting results for the expression of (A) SET7/9 (B) SET1A (C) SET1B (D) MLL1 (E) MLL2 and (F) MLL4 of HPMCs. GAPDH was used as an internal control. Lower panel: quantification. Data are means ± S.D. *, P < 0.05 (Student’s t test; n = 5 samples per group). (TIF) [file pone.0196844.s007.tif]

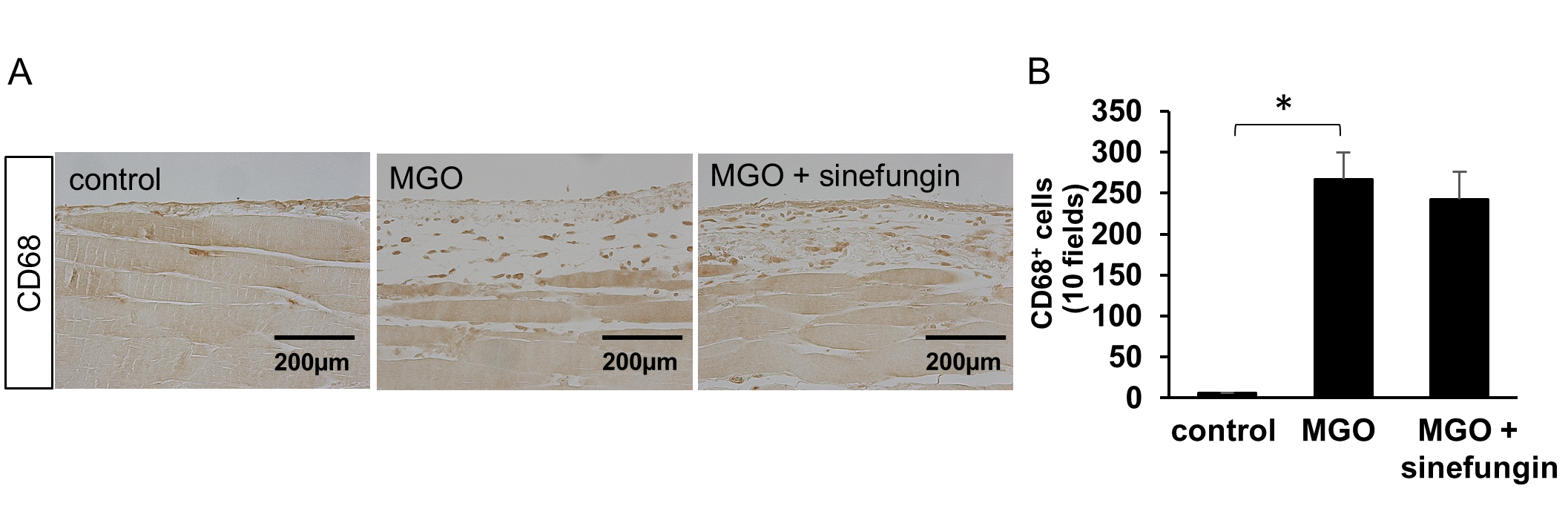

Supplement: S8 Fig — (A) Typical CD68 expression in peritoneal tissues of control mice, MGO-injected mice treated with vehicle only and MGO-injected mice treated with sinefungin (immunohistochemical [IHC] stain, ×200). (B) Numbers of CD68-positive (CD68+) cells in the 3 groups of mice. Scale Bar = 200 μm. Data are means ± S.D. *, P < 0.05 (one-way ANOVA followed by post hoc test using t test with Bonferroni correction; n = 5 mice per group). (TIF) [file pone.0196844.s008.tif]
